# Visual disgust constricts pupils in response to misophonic movies

**DOI:** 10.3389/fpsyg.2025.1569598

**Published:** 2025-08-05

**Authors:** Yuqi Qiu, Sungjoon Park, Urszula Oszczapinska, Laurie M. Heller

**Affiliations:** ^1^School of Computer Science, Carnegie Mellon University, Pittsburgh, PA, United States; ^2^Department of Psychology, Carnegie Mellon University, Pittsburgh, PA, United States; ^3^Center for the Neural Basis of Cognition, Pittsburgh, PA, United States

**Keywords:** misophonia, disgust, auditory-visual, pupillometry, everyday sounds, cognition, emotion, reappraisal

## Abstract

Misophonia is a condition typically described as heightened intolerance to specific everyday sounds, although intense emotional and physiological responses can also be triggered by non-auditory representations of the sources of these sounds, e.g., words, videos, or imagination (Swedo et al., 2022). We asked whether pupillometry could provide an objective pupillary signature of the reactions of disgust and anger toward misophonic events depicted in movies. We found greater pupil constriction toward movies with more visually disgusting video tracks, both for misophonic and non-misophonic individuals, whereas movies with a video track suggesting a neutral source of a misophonic sound (e.g., Samermit et al., 2022, Heller et al., 2025) increased both the sound pleasantness ratings and the pupil diameters. Furthermore, repeated exposure to the same sounds in different movies changed pupil responses such that they diverged from the ratings of sound unpleasantness. The findings of this study may provide valuable insights into the understanding, diagnosis, and treatment of misophonia.

## 1 Introduction

Misophonia is a neurophysiological and behavioral condition characterized by a heightened intolerance to certain stimuli, commonly referred to as “triggers” (Ferrer-Torres and Giménez-Llort, 2022; Swedo et al., 2022). Triggers are predominantly human-produced sounds, such as chewing, slurping, gulping, and finger tapping, which elicit intense physical and emotional responses regardless of their loudness (Ferrer-Torres and Giménez-Llort, 2022; Hansen et al., 2021; Jastreboff and Jastreboff, 2001). Individuals with misophonia exposed to triggers commonly experience increased muscle constriction and heart rate (Edelstein et al., 2013), as well as emotional reactions, including feeling unbearable levels of anger, anxiety, and disgust (Swedo et al., 2022). Severe presentations of misophonia can greatly affect quality of life and contribute to a higher risk of depression (Dibb and Golding, 2022). Although most research on misophonia focuses on auditory triggers, studies have found that visual stimuli associated with misophonic sounds can elicit similar responses (Ferrer-Torres and Giménez-Llort, 2022). Furthermore, Ferrer-Torres and Giménez-Llort (2021) found that merely imagining misophonic sounds can trigger similar reactions. These findings suggest that misophonic responses may extend beyond auditory stimuli. However, it remains unclear whether the effects observed in visual studies are driven solely by the visual stimuli themselves or by the conceptualization of sounds within a visual context.

Previous studies on the general population have shown that emotional sounds elicit physiological reactions, including changes in heart rate, heart rate variability, and/or skin conductance, with some studies finding an increase in physiological responses (Khalfa et al., 2002; Jürgens et al., 2018) and others finding a decrease or a mix of increases and decreases (Bradley and Lang, 2000; Brouwer et al., 2013; Dimitriev et al., 2023; Lundqvist et al., 2009). These studies often categorize sounds based on general valence (positive, negative, and neutral emotions), with findings suggesting that negative stimuli tend to elicit stronger physiological reactions. Using emotional visual stimuli, research has found increased skin conductance and decreased heart rate when participants view both unpleasant and pleasant images (Bernat et al., 2006; Bradley et al., 2008; Sánchez-Navarro et al., 2006; Winton et al., 1984). However, Winton et al. (1984) also observed that the heart rate only decreases in response to unpleasant images, while it increases in response to pleasant images. These mixed findings make it difficult to predict physiological responses based on the emotional valence of unimodal auditory or visual stimuli.

Ekman et al. (1983) found that physiological responses can not only distinguish between positive, negative, and neutral emotions, but also between specific negative emotions. Herein, we focus on physiological responses to disgust and anger because of their relevance for misophonia. For example, Vrana (1994) examined multimodal responses (auditory and visual) to disgust, anger, and neutral imagery by measuring startle reflex, heart rate, and skin conductance. They found an increase in startle reflex and heart rate when participants viewed disgusting or anger-inducing images, but did not observe differences in skin conductance. However, this study was unable to distinguish the specific effects of disgust and anger. Another study by Rohrmann and Hopp (2008) showed movies that described amputation and vomiting, both of which evoked strong feelings of disgust. While vomit movies caused a significant increase in heart rate, amputation movies resulted in a decrease in heart rate, possibly due to fear-bradycardia (slowing of heart rate due to fear). Skin conductance also increased when watching these disgusting movies, in contrast with Vrana (1994). The inconsistency of findings suggest that these physiological measures are not yet known to be reliable indicators of misophonic emotions.

In addition to heart rate and skin conductance, pupillometry has been utilized as an objective measure to record states of arousal by tracking pupil dilation and constriction in response to stimuli. Pupils are regulated by both the sympathetic nervous system (SNS) and the parasympathetic nervous system (PNS). The PNS typically engages with pupil constriction from increased brightness (Szabadi, 2018), while the SNS correlates with pupil dilation from emotional responses (Widmann et al., 2018). The pupil size is decided by the balance between the PNS and the SNS (Zekveld et al., 2018). When a visual object appears, there is a pupillary light reflex, an automatic response in which the pupil rapidly constricts to minimize the amount of light the eye receives (Bradley et al., 2008; Ferrari et al., 2016; Snowden et al., 2016). After the initial brightness adjustment, the diameter of the pupil reflects the influences of emotional responses. Studies show that pupil dilation increases with emotional arousal across the valence spectrum (Bradley et al., 2008; Kassem et al., 2017; Laeng et al., 2012; Partala and Surakka, 2003; Wang et al., 2018a). In addition, factors such as moderate brightness, increased cognitive activity, greater loudness, and lower fatigue have also been associated with maximal pupil dilation (Elshtain and Schaefer, 1968; Granholm et al., 1996; Steinhauer et al., 2004; Wang et al., 2018b; Zekveld et al., 2018).

Studies have found pupil dilation to both highly arousing auditory stimuli (e.g., a baby crying or laughing) (Partala and Surakka, 2003; Partala et al., 2000) and highly arousing visual stimuli (e.g., picture of a bloody hand) (Bradley et al., 2008; Murokawa and Nakayama, 2021; Henderson et al., 2018). In particular, Smith et al. (2025) found that people with tinnitus and auditory hypersensitivity disorders, including misophonia, exhibit significant pupil dilation when listening to emotionally evocative sounds, ranging from highly unpleasant (e.g., unpleasant animal sounds) to highly pleasant (e.g., peppy music), consistent with prior observations that misophonic individuals experience increased physiological responses to emotional triggers. Furthermore, Nakakoga et al. (2020) investigated emotional responses to visual and auditory stimuli separately and found asymmetric pupillary responses to the two. Their study consisted of two parts: in the first, participants viewed pictures, and in the second, participants listened to sounds. During each sensory study, the stimuli were conditioned to different attentional states: emotional tasks (evaluating the pleasantness of the stimuli), visual tasks (detecting a dot on the screen), auditory tasks (detecting a beep sound), and no tasks. Across all eight stimulus and task conditions, they observed a consistent pupil dilation response. Moreover, in the visual study, the pupils dilated more rapidly to both positive and negative images, while in the auditory task, the pupils dilated faster for neutral and positive sounds. The observed asymmetry in pupillary responses to visual vs. auditory stimuli suggests a potential difference in the way that the brain processes emotional stimuli from these two modalities. The authors speculate that auditory responses may be influenced by attention, while visual responses may be less affected by attention.

However, there have been exceptions to the generalization that pupil dilation increases with emotional intensity. Two empirical studies have shown a negative correlation between visual disgust levels and pupil size, indicating a constriction response. Schienle et al. (2016) explored pupillary responses to pictures that evoke disgusting, fearful, or neutral emotions. These researchers found that only disgust ratings negatively correlated with pupil sizes. Another study examined trypophobia, a condition characterized by negative emotional responses triggered by exposure to images containing “holes” (Ayzenberg et al., 2018). Unlike certain phobias that primarily elicit fear responses, trypophobia also evokes disgust. The latter study found a pattern of pupil constriction related to disgust, which aligns with PNS activation, but not constriction for fear or neutral conditions. This agrees with behavioral studies which found that people's eyes naturally narrow to express emotions of disgust (Lee et al., 2014). While previous studies have examined the potential impact of visual or conceptual disgust on pupil sizes, fewer have looked at purely auditory disgust (Oszczapinska et al., 2025) or auditory aversiveness (De Gee et al., 2024) and none have explored auditory-visual disgust while distinguishing it from fear and/or anger. Both disgust and anger are commonly listed as key components of misophonia. Swedo et al. (2022), in their Consensus Definition of Misophonia, stated that “individuals with misophonia may experience a range of negative affective reactions. Anger, irritation, disgust, and anxiety are most common, though some individuals may experience rage.” Furthermore, having higher disgust sensitivity increases the risk of having misophonia, although it is not a trait of every misophonic individual (Barahmand et al., 2023; Olatunji et al., 2009; Schröder et al., 2013; Taylor, 2017).

In general, pupillometry is useful for gauging emotional intensity but does not separate different emotions, as both positive and negative emotions tend to increase pupil diameter. The one exception is visual disgust, which has sometimes been shown to decrease pupil diameter across a variety of stimuli such as movies (Lee et al., 2023), sounds and pictures (McCulloch et al., 2025), and facial expressions (Prunty et al., 2022). Because anger is expected to increase pupil diameter, while disgust is expected to decrease pupil diameter, we aimed to find a signature of disgust through pupillometry while measuring ratings of both anger and disgust toward the stimuli to discover which emotion had more explanatory power. Therefore, our first aim is to explore the relationship between pupil size and audio-visually evoked emotions. Furthermore, because the physiological effects of visual stimuli on the response to unpleasant auditory stimuli are not well understood in general, we tested both misophonic and non-misophonic populations (Polo et al., 2022).

A second aim of this paper is to understand how contextual understanding of sounds, i.e., when the perceived unpleasantness of misophonic sounds is altered by visual stimuli, is reflected in pupil responses. Previous studies of misophonia using auditory-visual stimuli found that neutral-to-pleasant visual stimuli paired with misophonic sounds (e.g., pairing the misophonic sound of a person eating crunchy chips with the neutral video of a person shaking beads in a plastic bottle) could lead to a reinterpretation of the source of the sounds, thus influencing individuals' perceptions of the sound and its overall pleasantness (Samermit et al., 2022; Heller et al., 2025). Mahzouni et al. (2024) also measured bodily sensations in addition to pleasantness ratings, and found that while both the misophonic and control groups had increased pleasantness ratings when trigger sounds were paired with more positive visual contexts, only the misophonic group showed a larger reduction in bodily sensations. However, responses to such audiovisual stimuli have not been related to pupillometry. It is not currently known how to predict pupil dilation vs. constriction for such audiovisual stimuli. As reviewed above, while an unpleasant sound is predicted to dilate pupils (Oszczapinska et al., 2025), a disgusting visual stimulus is predicted to constrict pupils. Therefore, which unimodally-specified effect dominates in an auditory-visual context needs to be empirically tested.

We therefore hypothesized that pupil diameters would be larger when novel misophonic sounds are accompanied by video tracks suggesting neutral sources than when accompanied by unpleasant video tracks depicting the true sound source. The order in which visual context is presented to the participant is expected to be important, as previous research has reported an order effect, showing a greater pleasantness difference when the neutral context is shown first compared to the unpleasant context (Samermit et al., 2022). Furthermore, we predicted a larger emotional and physiological response for misophonic individuals, reflecting their higher sensitivity to those sounds. Although the misophonic stimuli may be merely “unpleasant” rather than “triggering” to non-misophonic participants, we aim to encompass the responses across both populations by studying unpleasantness and disgust.

If movies suggesting neutral reappraisal of misophonic sounds were to be used in a therapeutic setting, they might be presented multiple times to the same individual. An underexplored area in misophonia research is how emotional and physiological responses change through repeated exposure to trigger sounds, or to their visual counterparts. Thus, a third aim of this paper is to test whether repeated exposures to the same sound in different visual contexts, as well as repetition of auditory-visual pairs, produce consistent effects on both pupil diameters and perceived pleasantness. Repetition could, in principle, cause different phenomena which have opposing consequences: habituation, sensitization, or the mere exposure effect. Habituation is a phenomenon in which the magnitude of physiological and emotional responses to a stimulus decreases with repetition (Bradley et al., 1993; Codispoti et al., 2006; Rankin et al., 2009). Based on habituation, one would expect the magnitude of pupillary responses and ratings to everyday neutral and unpleasant sounds to decrease with repeated exposures. However, evidence suggests that individuals with misophonia do not habituate to repeated trigger sounds. Instead, their responses intensify with time, possibly indicating sensitization rather than habituation (Frank and McKay, 2019; Schröder et al., 2019). Similarly, increased sensitivity has been observed over time in people with OCD who were repeatedly exposed to disgust-evoking tasks (Olatunji et al., 2009). Furthermore, the *mere exposure effect* (Zajonc, 1968) suggests that repeated exposure to neutral or mildly positive stimuli leads to increased preference. Thus, one might expect sound pleasantness to increase with sound repetition. However, previous research shows that negative stimuli do not become more pleasant with repetition (Brickman et al., 1972; Burgess and Sales, 1971; Heller et al., 2025; Schröder et al., 2019). Therefore, varying phenomena suggest different outcomes for the effect of repetition of unpleasant sounds on pupil responses and/or perceived pleasantness. This study aimed to provide more evidence for the nature of the relationship between repetition of neutral and unpleasant sounds and pupillary and emotional responses for both misophonic and non-misophonic individuals.

It is also of interest to assess the interaction between visual context and repeated exposure. The video component of a movie can serve as a way to change or confirm the source of the audio. We hypothesize that when an unpleasant sound is repeated in a movie with an unpleasant video after initial exposure in a neutral movie, reappraisal of the sound will, if anything, decrease sound pleasantness ratings. Conversely, for repeated exposure to a sound in a neutral movie after initial exposure to an unpleasant movie, one might logically expect reappraisal to increase sound pleasantness ratings. However, this expectation is moderated by prior studies, Samermit et al. (2022) and Heller et al. (2025), which found that the intensity of emotional ratings of sounds decreases after seeing more than one source interpretation. That is, an unpleasant sound heard in the context of a neutral movie is more positive if it has never been heard in the context of its original unpleasant video track. Therefore, we predict that the emotional response to the sounds will be closer to neutral for all sounds that have been seen in opposite movie conditions. For example, after a participant watches an unpleasant movie, their mildly positive emotional response to a neutral-pleasant movie with the same sound would be smaller than if they saw the neutral movie before seeing the unpleasant movie.

Together, we assessed these hypotheses using both physiological (pupil measurements) and behavioral (ratings) data. Using a passive viewing paradigm, we measured pupil reactivity to unpleasant sounds when they were viewed in the context of both neutral and unpleasant visual events that each provide a unique causal explanation for the sound. Sound pleasantness ratings were obtained after viewing each movie. In separate studies, we measured unimodal emotional ratings of disgust, anger, and fear for the visual and auditory components of the movies. We ask whether there are differences in pupil reactivity and/or emotional reactivity to these movies for people with and without misophonia. In addition, we ask whether there are differences in pupil reactivity and/or pleasantness ratings across repeated exposure. Monitoring changes in pupil size over time may provide valuable information for identifying misophonic patients and/or evaluating the effectiveness of treatments that aim to reduce the unpleasant emotional responses associated with misophonic triggers.

## 2 Experiment 1

Experiment 1 was multi-pronged. First, we assessed the difference in pupil size to unpleasant sounds paired with their original, unpleasant video tracks (depicting the true sound source) compared to when they were paired with alternative, neutral video tracks depicting alternative sound sources. We also examined how pupil size changes with repeated exposure. Next, the changes in pupil size were correlated with sound pleasantness ratings, provided by participants after watching each movie. The goal was to understand the relationship between pleasantness and pupil response in different movie conditions, and to assess how the reinterpretation of sound from movie conditions can influence pleasantness ratings and pupil sizes.

### 2.1 Methods

#### 2.1.1 Participants

We recruited 65 participants (*M*_*AGE*_ = 24.6, *SD* = 5.9; 38 females, 24 males, 2 non-binary, 1 undisclosed) from Carnegie Mellon University and its surrounding area. Participants were between the ages of 18 and 41 due to known differences in pupillary reactivity in adult populations older than 45 (Lobato-Rincón et al., 2014). No participants were excluded based on race, ethnicity or gender. While we did not collect race and ethnicity data, the sample likely reflects Carnegie Mellon University's 2024 student population: 48.7% Asian, 41.1% White, 12.3% Hispanic, 7.8% Black, 0.8% Native American, 0.4% Pacific Islander, and 6.9% Other^1^ (Total percentage exceeded 100% due to duplicate counts). Prior to participating in the study, participants completed a set of scales that evaluated sound sensitivity, including the Duke-Vanderbilt Misophonia Screening Questionnaire (DVMSQ) Williams et al. (2022), the Misophonia Questionnaire (MQ) (Wu et al., 2014), and the S-Five (Vitoratou et al., 2023). All participants reported having normal hearing and normal-to-corrected vision. A subset of 26 participants scored at the clinically significant level on the DVMSQ, MQ, and/or S-Five, using the established clinical cutoffs for each scale, following prior research: meeting criteria A-E on the DVMSQ, a score of ≥7 on the Misophonia Severity Scale from the MQ, and ≥87 out of 250 on the S-Five. We found some agreement across these three misophonia classification standards: 22 participants met the misophonia threshold on the DVMSQ, 19 on the MQ, and 8 on the S-Five. Given this overlap and our a priori decision, we classified participants as misophonic if they met the threshold on any of the three criteria. For these 26 individuals, we conducted informal audiograms and measured loudness discomfort levels (LDL/ULL) (with the exception of 5 who were not tested due to procedural limitations). The difference in mean age between misophonic (*M* = 25.9, *SD* = 5.7) and non-misophonic (*M* = 23.6, *SD* = 5.8) observers was not significant [*t*_(54.93)_ = −1.28, *p* = 0.12]. All participants gave their informed consent and were compensated in cash according to an approved protocol of the CMU Institutional Review Board.

Audiograms and LDL/ULL measurements were administered to misophonic participants using a MAICO MA41 system that evaluated hearing sensitivity up to 8 kHz. The audiogram confirmed that 19 participants had normal hearing up to 8 kHz, as defined by ASHA (Type, Degree, and Configuration of Hearing Loss, 2023), with hearing thresholds below 20 dB HL. The remaining two participants demonstrated normal hearing up to 2 kHz. Additionally, all but one of our misophonic participants exhibited signs of hyperacusis, according to the LDL criteria defined by Sherlock and Formby (2005); Vidal et al. (2022) and using the procedure recommended by the British Society of Audiology (1987).

#### 2.1.2 Stimuli

We selected 28 of the 44 movies from the stimulus database described in Heller et al. (2025)^2^ and 2 movies from the Sound-Swapped Video Database described in Samermit et al. (2022). The database described in Heller et al. (2025) contains 44 movies of 22 unpleasant sounds (U_S_) paired with two different visual contexts: (1) its original, unpleasant visual context (U_V_, the unpleasant video track of the movie), and (2) a neutral visual context that provides an alternative source producing the unpleasant sound (N_V_, the neutral video track of the movie). The pairing of Us with the two visual contexts produced two types of movies: U_S_U_V_ and U_S_N_V_. For example, we paired the sound from a movie of a person sniffing repeatedly (*U*_*S*_*U*_*V*_) with a neutral video of a person pulling facial tissues from a tissue box (*U*_*S*_*N*_*V*_). In that previous study, each unpleasant sound received a pleasantness rating in both U_S_U_V_ and U_S_N_V_ movies by individuals with and without misophonia. To select videos for this study, we rank-ordered the 22 sounds by the magnitude of the change in sound pleasantness (U_S_N_V_−U_S_U_V_) when paired with its original unpleasant video track vs. its neutral alternative. This method ensured that the top-ranked movies were paired with the most “effective” neutral movies. The ranking was performed separately for average ratings of misophonic and non-misophonic listeners. Then, we took the top 10 ranked sounds from either misophonic or non-misophonic participants, which yielded 14 total sounds. Table 1 comprises the final 14 pairs of movies we presented. The loudness of the sounds was rms-equalized using custom code to control its impact on pupil size (Zekveld et al., 2018).

**Table 1:** The fourteen pairs of movies presented to participants, with paired movies having identical unpleasant sounds.

**Unpleasant video track condition (U_S_U_V_)**	**Neutral video track condition (U_S_N_V_)**	**Database of sounds**	**Database of video tracks**
Person smacking lips	Pulling sticky tape from a tape dispenser	Lab recording	Lab recording
Person sucking air in through their teeth	Pulling measuring tape from a tape dispenser	Lab recording	Lab recording
Person sniffing in long intervals	Pulling napkin from a napkin box	Lab recording	Lab recording
Person sniffing in short intervals	Brush brushing against a table	Lab recording	Lab recording
Person brushing their teeth	Sprinkler on a lawn	Lab recording	Lab recording
A plastic bottle being crinkled	Fire crackling	Lab recording	U_S_U_V_ from lab recording, U_S_N_V_ from YouTube
Person eating crunchy chips	Shaking beads in a plastic bottle	Lab recording	Lab recording
Person gulping water	Jug bubbling water	Lab recording	Lab recording
Person wheezing	Pressing down on an air pump	Lab recording	Lab recording
Metal utensils scraping against each other	Birds chirping	Lab recording	U_S_U_V_ from lab recording, U_S_N_V_ from YouTube
Person swishing water in their mouth	Duck swishing in water	Lab recording	Lab recording
Nails scratching against a blackboard	Tearing apart fabric	Lab recording	Lab recording
Fingertips tapping against a table	Ball bouncing on a table	Sound–swapped Video database	Sound–swapped video database
Person slurping a drink through a straw	River flowing	Sound–swapped video database	Sound–swappedvideo database

All movies were made equal in length by including only the first 8 s. Next, to control for image brightness, we converted the movies to grayscale in iMovie ^3^ and normalized brightness to 40 cd/m^2^ luminance using custom Matlab scalar code. These adjustments were verified with a TES 137 light meter (± 3 cd/m^2^). Additionally, the spectral frequency of each movie was measured using custom Python code. Given that past studies showed that pupils constrict more when images have greater energy in the range of 2–8 cycles/deg (Hu et al., 2019), we compared the mean spectral energy in these ranges between the 14 U_S_U_V_ (X-axis: *M* = 6,407,849, *SD* = 62,807; Y-axis: *M* = 7,342,986, *SD* = 62,368) and the 14 U_S_N_V_ (X-axis: *M* = 6,479,184, *SD* = 145,794; Y-axis: *M* = 7,436,132, *SD* = 179,951) and found that they were not significantly different based on a Wilcoxon rank-sum test. Given that higher energy in these regions produces more pupil constriction, the non-significant mean difference between conditions could only produce greater constriction in the U_S_N_V_ condition, which is the opposite of our prediction.

To mitigate the pupillary reflex produced at the onset of a visual object on the display screen, we implemented a scrambled image procedure as a baseline control condition^4^. The objective was to match the brightness of the upcoming movie while removing semantic content. To generate these scrambled images, we first selected the frame in the middle of each movie using custom Python code. Then, we divided the frame into groups consisting of three adjacent lines of pixels. Within each group, we randomized the order of the pixels and reconstructed the scrambled frames accordingly. This process ensured that the scrambled images served as neutral stimuli with texture but without meaningful visual information, thus isolating the effects of semantic content on the pupil response and allowing the pupil size to adjust to a baseline level following pupillary reflex.

We then prepended each 8-s movie with its corresponding 2-s scrambled frame and appended a 1-s freeze of the final frame. The resulting 11-s clips were the stimuli in the experiment.

#### 2.1.3 Procedure

Movies were presented with a custom Matlab program (The MathWorks, Inc.) running on a 14-inch Dell monitor (Latitude 5,421, 1,920 × 1,080 pixels, 60 Hz refresh rate). Participants viewed 28 movies, each presented twice in random order, over a single 40-min session. The testing took place in a sound-attenuated chamber with a mean ambient luminance of 66 Lux. Participants were seated approximately 60 cm from the screen, and audio was played through Koss UR20 over-ear headphones at 60.5 dB SPL. The pupil sizes were recorded at 30 Hz using the SmartEye Aurora eye tracker (Version SE XO v9.3), which offers precise tracking without requiring a chin rest.

**Figure 1:**
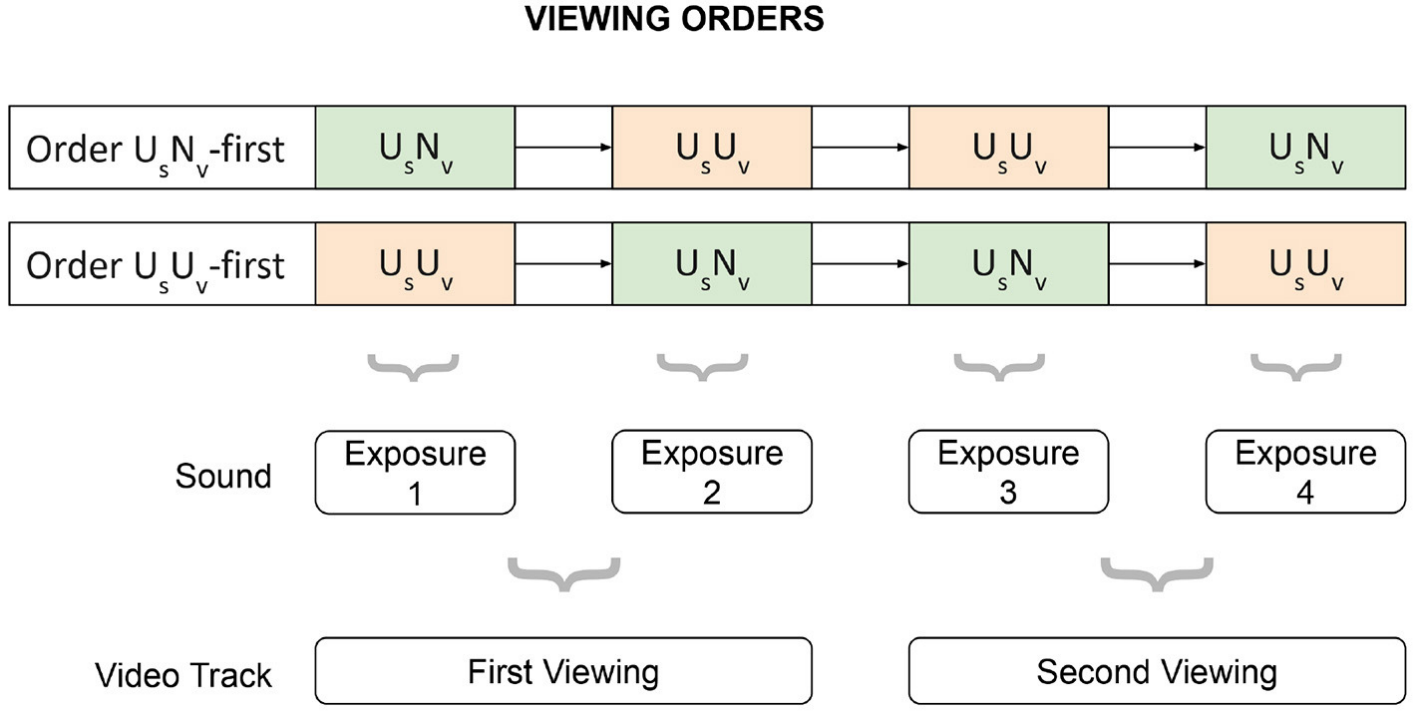
Presentation order of movies.

Participants were assigned to order U_S_N_V_-first (*N*= 41, 13 misophonic, 28 control) or order U_S_U_V_-first (*N* = 24, 13 misophonic, 11 control). In order U_S_N_V_-first, participants viewed all U_S_N_V_ movies followed by U_S_U_V_ movies during the initial viewing in the first half of the study, and this order was reversed for the second viewing in the second half of the study. Conversely, in order U_S_U_V_-first, participants watched U_S_U_V_ movies before U_S_N_V_ movies during the initial viewing, and reversed for the second viewing, The presentation order of movies within each condition (U_S_N_V_, U_S_U_V_) was randomized to minimize potential order effects. See Figure 1 for a visual representation.

Prior to beginning the experimental trials, each participant first completed a calibration test. During calibration, the participant fixated their gaze on four different locations bounding the image-displaying region of the screen. The gaze behavior during the calibration phase was compared with the gaze behavior during experimental trials to confirm that participants were watching the movies during the experiment. Following the calibration phase, participants read through on-screen instructions for the study and completed one practice trial with a research assistant in the room. Each trial began with a black fixation cross displayed in the center of a gray screen (RGB: 108, 108, 108; mean luminance = 40 cd/m^2^), upon which the participants were instructed to maintain their gaze. The fixation cross appeared for 2 s prior to the presentation of the 11-s stimuli. Then, participants rated the pleasantness of the sound in the movie on an 11-point Likert scale from 0 to 10, with -5 representing a very unpleasant sound, 0 representing a neutral sound, and +5 representing a very pleasant sound (Heller and Smith, 2022). After giving a rating, they pressed the “SPACE” key to advance to the next trial.

#### 2.1.4 Pupillary response pre-processing

Pupil data were pre-processed using a custom MATLAB program. We removed blinks and artifacts, as inspired by previous literature (Hershman et al., 2018) and closely modeled after (Oszczapinska et al., 2025). Following this step, we excluded trials for which there was no variability in pupil diameter. We proceeded to remove all trials in which at least 50% of the data had been removed in previous steps. We defined baseline as the average of all samples obtained 500 ms prior to the start of each movie, not including the scrambled image stage. Trials that did not contain any valid baseline data were excluded from further analysis. For the purposes of Figure 1, we performed linear interpolation to fill in gaps within the data, but for data analysis, no interpolation was done; only valid samples were averaged. We ensured that each participant had a valid pupil response for each pair of movies with the same sounds within each half of the experiment. For example, if pupillary responses were missing for the movie of “Person smacking lips” during the first (initial) movie viewing, then we excluded pupillary responses to the movie of “Pulling sticky tape from a tape dispenser” movie from the first movie viewing. We applied the same exclusion criteria for subsequent movie viewings. This equated the sounds across both visual conditions per person to avoid unbalanced data due to differences between sounds or individuals. Finally, to examine pupillary changes across time for each movie, we subtracted pupil diameter at baseline from pupil diameter while individual movies were viewed at the participant level. Therefore, all reported pupil measures indicate the change from baseline induced by the movie. For all analyses, pupil diameter was averaged from 1.5 to 6 s after the onset of each 8-s movie (i.e., excluding the initial 2-s scrambled image and excluding the initial 1.5 s of the movie, and ending 2 s before the movie ended to avoid any eye movements related to anticipating the need to respond toward the end of the movie).

### 2.2 Results

#### 2.2.1 Pupillary data analysis

To analyze the physiological changes evoked by the movies, we conducted two primary analyses:

##### 2.2.1.1 Analysis 1: first exposure

**Figure 2:**
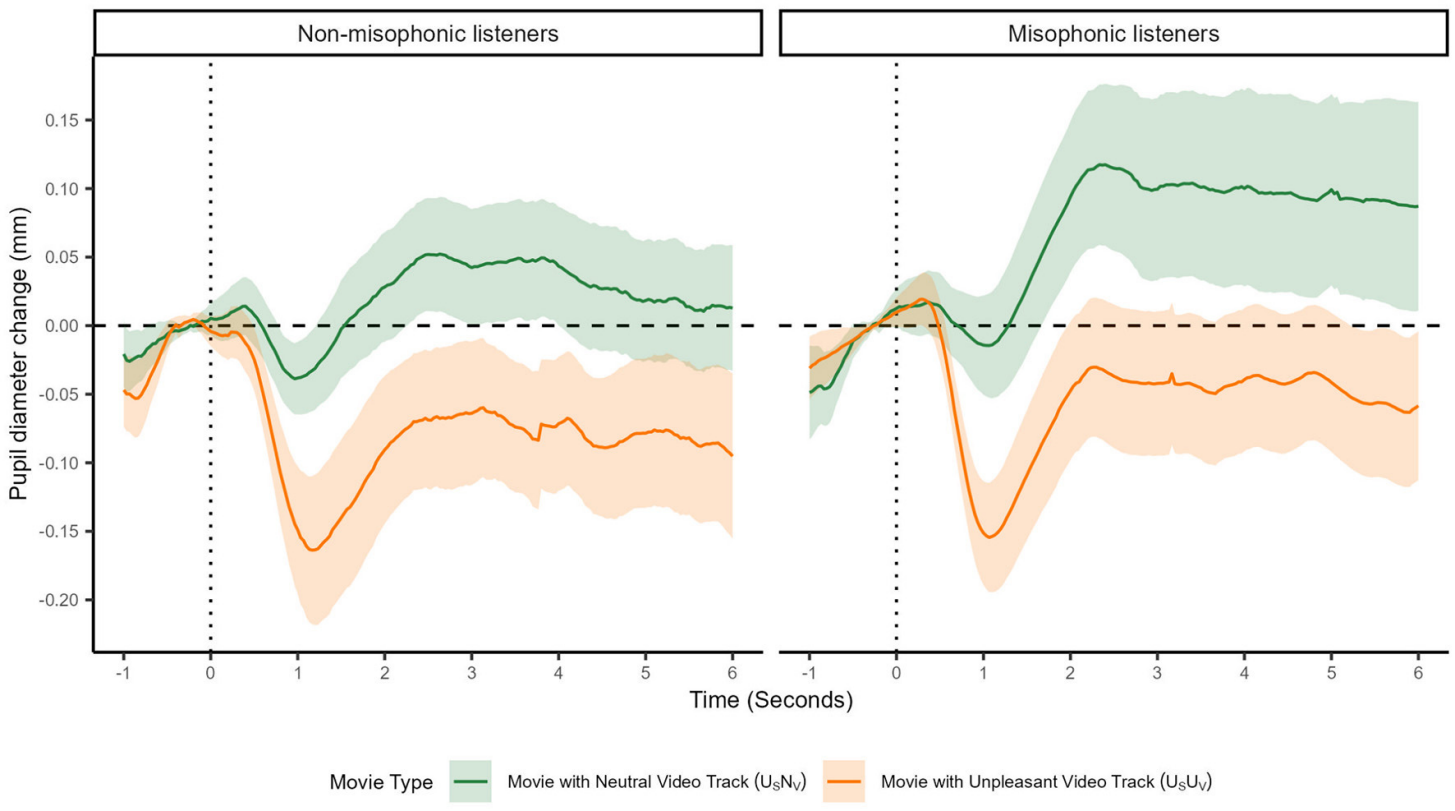
Sound-averaged pupil diameter over the course of a movie during the first exposure to a sound. The first exposure to the sound was within a movie paired with either an Unpleasant or Neutral video track, depending upon the test order. The x-axis represents movie time (in seconds) and the y-axis represents the pupil diameter change from baseline (in millimeters). The shaded regions indicate 95% confidence interval. U_S_U_V_ is displayed in orange and U_S_N_V_ is displayed in green. The left and right panels show means for non-misophonic and misophonic participants, respectively.

We focused on participants' first exposure to each sound; we compared pupillary changes toward U_S_N_V_ (unpleasant sound with neutral video track) and U_S_U_V_ (unpleasant sound with unpleasant video track) conditions for sounds. We also compared ratings of each sound from misophonic and non-misophonic groups (Figure 2). Note that the first exposure corresponds to the U_S_N_V_ condition for participants with test Order U_S_N_V_-first, and corresponds to the U_S_U_V_ condition for participants with test Order U_S_U_V_-first.

To isolate the effects of the first exposure to sound, this analysis uses a 2x2 between-subjects design, separating participants based on their Misophonic status (misophonic or non-misophonic) and Movie type (U_S_U_V_ or U_S_N_V_). An ANOVA was conducted across movies with factors of Misophonic status (misophonic or non-misophonic) and Movie type (U_S_U_V_ or U_S_N_V_). A significant main effect was observed for Misophonic status [*F*_(1, 13)_ = 4.729, *p* < 0.049, $\eta_{p}^{2}=0.267$], misophonic listeners produced slightly more positive pupil diameter changes relative to baseline [*U*_*S*_*N*_*V*_:*M* = 0.10 mm, 95% CI = (0.02, 0.18); *U*_*S*_*U*_*V*_:*M* = −0.05 mm, 95% CI = (-0.12, 0.02)] compared to non-misophonics [*U*_*S*_*N*_*V*_:*M* = 0.03 mm, 95% CI = (−0.03, 0.10); *U*_*S*_*U*_*V*_:*M* = −0.09 mm, 95% CI = (−0.14, −0.03)]. Additionally, we observed a main effect of Movie type [*F*_(1, 13)_ = 7.929, *p* < 0.015, $\eta_{p}^{2}=0.379$], evidenced by pupils reliably dilated from baseline by 0.07 mm [95% CI = (0.001, 0.132)] in response to movies containing neutral visual tracks and unpleasant sounds (U_S_N_V_). In contrast, pupils reliably constricted from baseline by −0.07 mm [95% CI = (−0.122, −0.014), $F_{\left(1,13\right)}=0.58,p=0.46,\eta_{p}^{2}=0.43$] in response to movies containing unpleasant visual tracks and sounds (U_S_U_V_). Misophonic status and Movie type did not interact [*F*_(1, 13)_ = 0.58, *p* = 0.46, $\eta_{p}^{2}\;=\;0.042$].

##### 2.2.1.2 Analysis 2: repeated exposures

**Figure 3:**
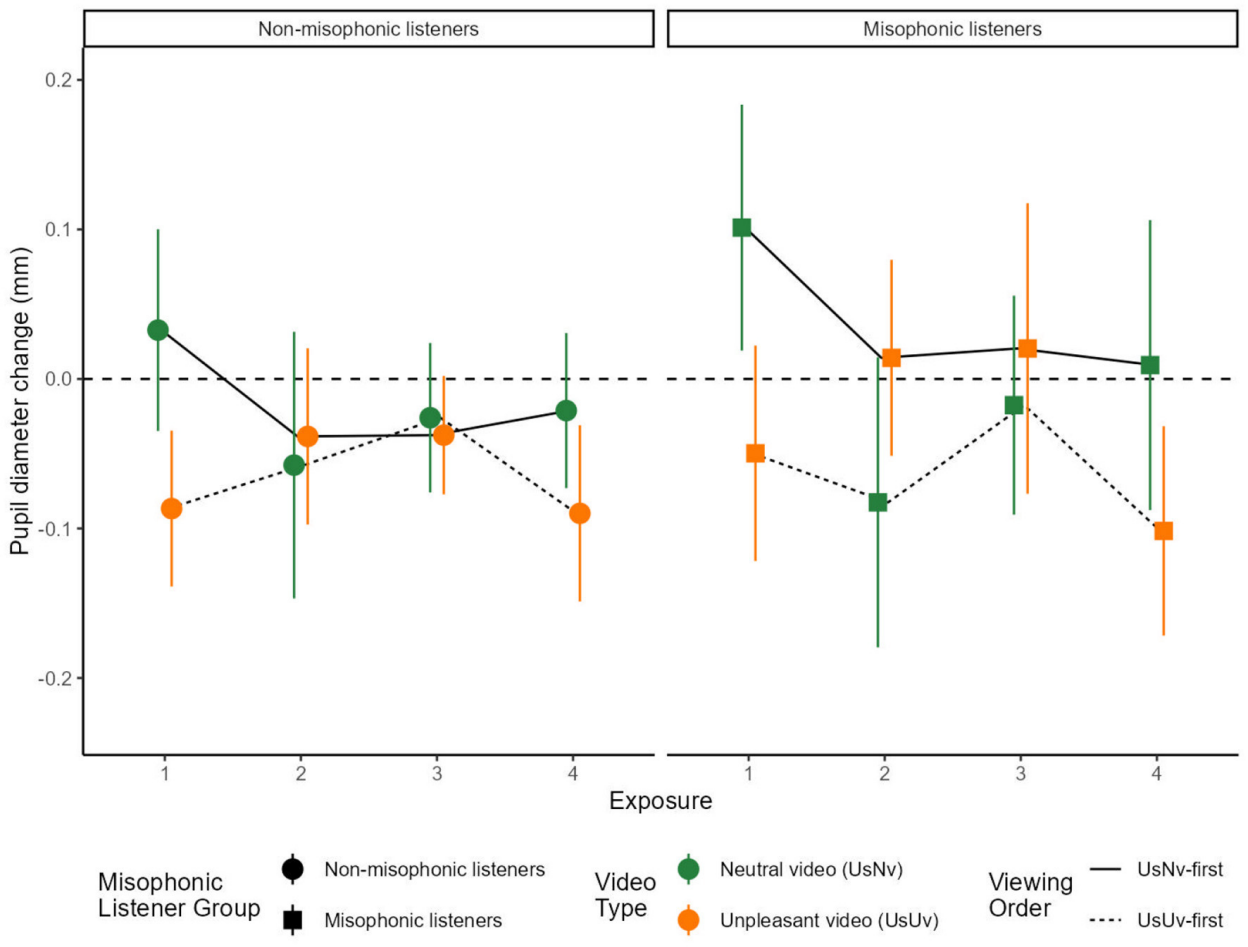
Mean pupil diameter over sound exposures. The x-axis represents the exposure number and the y-axis represents the average pupil diameter (in milliseconds) from 1.5 to 6 s. Error bars represent the 95% confidence intervals for each condition. U_S_U_V_ is displayed in orange and U_S_N_V_ is displayed in green. The dotted line traces the U_S_N_V_-first order condition, and the solid line traces the U_S_U_V_-first order condition. The data in this figure shows all four sound exposures for both order conditions.

We compared average pupil diameter for each movie between the U_S_N_V_ and U_S_U_V_ conditions across misophonic and non-misophonic participants (Figure 3). A 3-way ANOVA included factors of sound Exposure number (four exposures, as each sound was presented twice with both movie types—see Figure 1 in Methods), as well as Order and Misophonic status. There were significant main effects of Exposure number [*F*_(1, 39)_ = 4.13, *p* < 0.05, $\eta_{p}^{2}=0.24$], showing that average pupil diameter varied across exposures. There was a main effect of Order [*F*_(1, 13)_ = 22.99, *p* < 0.001, $\eta_{p}^{2}=0.64$], evidenced by greater average pupil diameter for the U_S_N_V_-first condition [*M* = 0.01, 95% CI = (−0.832, 1.08)] compared to U_S_U_V_-first condition [*M* = 0.01, 95% CI = (−0.832, 1.08)], and a main effect of misophonia status [*F*_(1, 13)_ = 5.76, *p* < 0.05, $\eta_{p}^{2}=0.24$], where non-misophonic participants tend to show greater pupil constriction [*M* = −0.041, 95% CI = (−0.068, −0.013)] compared to misophonic participants [*M* = −0.013, 95% CI = (−0.053, 0.026)]. We did not observe any significant interactions.

##### 2.2.1.3 Resampling analysis

To address the imbalanced number of non-misophonic participants between our test groups, all of our reported ANOVAs used averaged data per sound rather than per participant. Therefore, there was no confounding factor in the degrees of freedom across different test groups. To address potential concerns about this issue, we randomly subsampled a matching number of non-misophonics from the U_S_N_V_-first Order a total of 30 times. The median p-value of all 30 ANOVAs was taken. All of the full-sample ANOVA main effects and interactions were maintained. A similar resampling analysis was also conducted for all other ANOVAs which confirmed all significant effects reported for ratings and pupils in Analyses 1–4, with only one exception: the 3-way interaction in Analysis 4 presented in section 2.2.2 was significant in less than half of the samples.

Focused comparisons of same movie conditions. To understand the effects of initial cognitive appraisal and reappraisal on sounds through repeated exposures, we analyzed pupillary data for the same movies across groups in sound Exposures 1 and 2, followed by an analysis of the same movies within groups in sound Exposures 1 and 4.

We compared across order groups to ask whether the pupil response upon the initial *viewing* of the U_S_U_V_ movie depended on whether the sound was being *heard* for the first time (sound Exposure 1 in the U_S_U_V_-first order) or after a first exposure to the sound within the U_S_N_V_ movie (sound Exposure 2 in the U_S_N_V_-first order). In Figure 3, these two data points are indicated by the two leftmost orange symbols within each panel. For misophonic participants (right panel), the pupil diameter change from baseline for the initial viewing of the unpleasant video was more negative (more constricted) when it did not follow a neutral video (U_S_N_V_ Exposure 2 - U_S_U_V_ Exposure 1: *M* = 0.0638, *SE* = 0.028, *t*_(13)_ = 2.30, *p* < 0.038), whereas for non-misophonic participants (left panel), this pupil change was also less constricted but not significantly so (U_S_N_V_ Exposure 2 - U_S_U_V_ Exposure 1: *M* = 0.0483, *SE* = 0.026, *t*_(13)_ = 1.85, *p* = 0.088). In summary, a first exposure to an unpleasant sound in the context of a U_S_N_V_ movie had effects on misophonics but not on non-misophonics, resulting in a *less constricted* pupil diameter from baseline during the initial viewing of the U_S_U_V_ movie by misophonics.

In a parallel analysis for Exposures 1 and 2, we asked whether the pupil response upon the initial *viewing* of the U_S_N_V_ movie depended on whether the sound was being *heard* for the first time (Exposure 1 in the U_S_N_V_-first order) or after a first exposure to the sound within the U_S_U_V_ movie (Exposure 2 in the U_S_U_V_-first order). In Figure 3, these two data points are shown by the two leftmost green symbols within each panel. The pupil change from baseline of the initial neutral video condition was more constricting when it followed an unpleasant video condition for both misophonic participants (U_S_U_V_ Exposure 2 - U_S_N_V_ Exposure 1: *M* = −0.184, *SE*= 0.037, *t*_(13)_= 4.95, *p* < 0.0003), and for non-misophonic participants (U_S_U_V_ Exposure 2 - U_S_N_V_ Exposure 1: *M* = −0.090, *SE*= 0.032, *t*_(13)_ = 2.85, *p* < 0.014). In summary, a first exposure to an unpleasant sound in the context of a U_S_U_V_ movie has similar effects on misophonics and non-misophonics, resulting in a *more constricted* pupil diameter from baseline during the initial viewing of the U_S_N_V_ movie by both participant groups.

Next, we asked whether the pupil response was changed by prior viewing of alternative movies *within* participants. During sound Exposures 1 and 4, each participant saw the same movie either as the first exposure to the sound (which was also the initial viewing of any movie) or as a fourth exposure to the sound (which followed two intervening movies that displayed an alternative source for the sound). Because the type of movie being compared was within participant (U_S_U_V_ or U_S_N_V_), depending upon their test order, each test order was separately analyzed for misophonics and non-misophonics. We first analyzed just the group in the U_S_U_V_-first order. In Figure 3, these two data points are shown by the leftmost and rightmost orange symbols within each panel. We ask whether the participants' responses to the U_S_U_V_ movie differed between two repeated viewings: the initial (first) viewing presented in sound Exposure 1 (U_S_U_V_-first order), and the second viewing presented in sound Exposure 4 which follows two viewings of the neutral interpretation of the same sound in the U_S_N_V_ movie (U_S_U_V_-first order). For misophonic participants, the difference between these exposures was marginally significant [U_S_U_V_ Exposure 4 - Exposure 1: *M* = −0.052, *SE* = 0.025, *t*_(13)_ = 2.04, *p* = 0.062] but for non-misophonic participants, the mean difference was not significant [U_S_U_V_ Exposure 4 - Exposure 1: *M* = −0.003, *SE* = 0.029, *t*_(13)_ = 0.11, *p* = 0.91]. In summary, the pupil response to the first and fourth exposure of an unpleasant sound in a U_S_U_V_ movie was primarily determined by the movie context it was viewed in during that trial, because it was not significantly affected (despite some marginal effects) by prior viewing of intervening movies with alternative source interpretations.

In a parallel analysis for Exposures 1 and 4, we asked whether the pupil response to the neutral U_S_N_V_ movie differed between sound Exposures 1 and 4 within participants. We compared the initial viewing in Exposure 1 (U_S_N_V_-first order) to the second viewing in Exposure 4 (which follows two viewings of the unpleasant interpretation of the same sound in the U_S_U_V_ movie (U_S_N_V_-first order). In Figure 3, these two data points are shown by the leftmost and rightmost green symbols within each panel. The difference between these exposures is marginally significant for both misophonic participants [U_S_N_V_ Exposure 4 - Exposure 1: *M* = −0.092, *SE* = 0.048, *t*_(13)_ = 1.94, *p* = 0.075] and non-misophonic participants [U_S_N_V_ Exposure 4 - Exposure 1: *M* = −0.054, *SE* = 0.030, *t*_(13)_ = 1.79, *p* = 0.096]. In summary, the pupil response to the first and fourth exposure of an unpleasant sound in a U_S_N_V_ movie was primarily determined by the movie context it was viewed in during that trial, because it was not significantly affected (despite marginal effects) by prior viewing of intervening movies with alternative source interpretations.

#### 2.2.2 Behavioral data analysis

**Figure 4:**
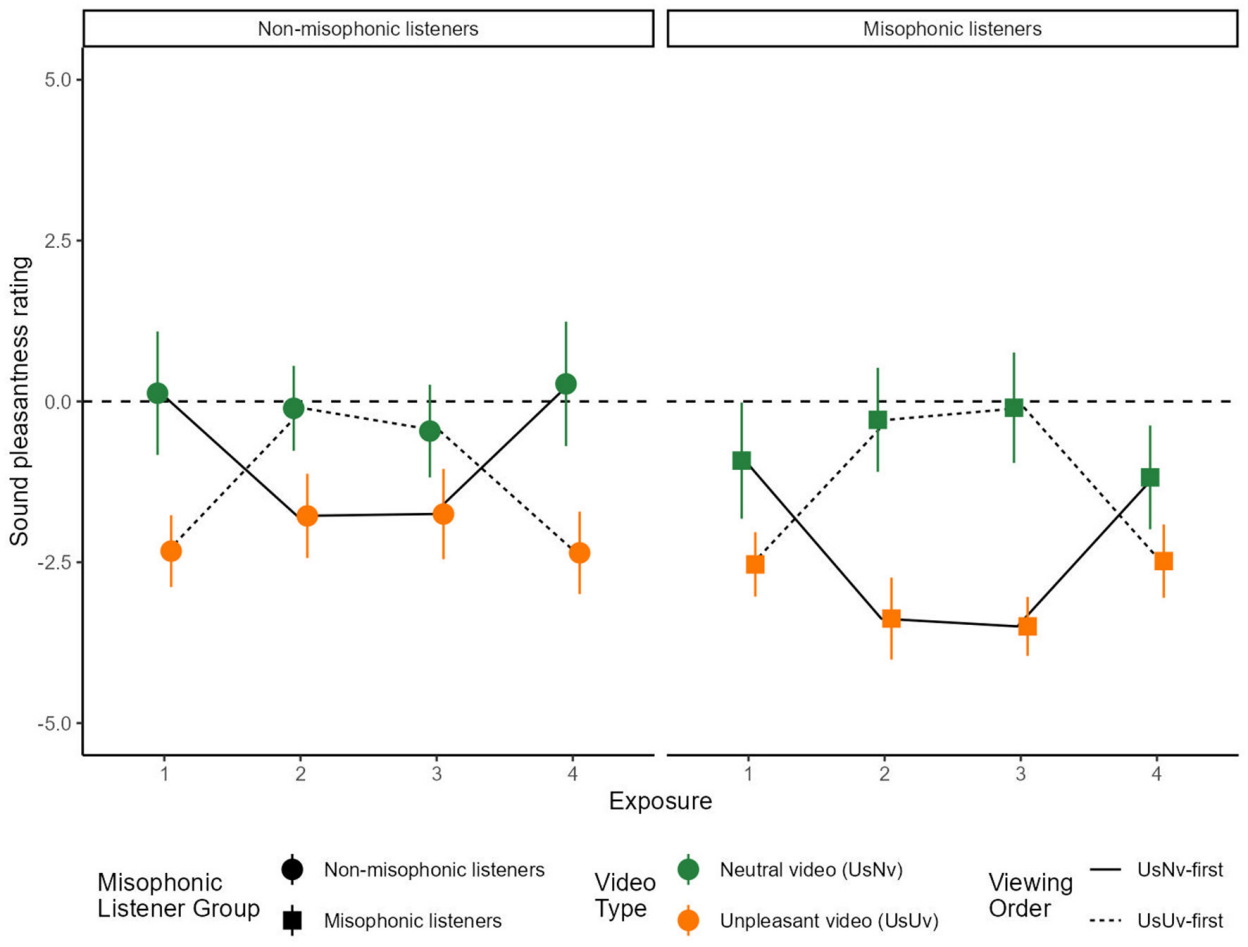
Mean sound pleasantness rating over sound exposures. The x-axis represents the exposure number and the y-axis represents the average sound pleasantness rating for each movie. Error bars represent the 95% confidence intervals for each condition. U_S_U_V_ is displayed in orange and U_S_N_V_ is displayed in green. The dotted line traces the U_S_N_V_-first order condition, and the solid line traces the U_S_U_V_-first order condition. The data in this figure shows all four sound exposures for both order conditions.

To analyze the emotional effects induced by the movies, we conducted two analyses of the rating data that were parallel to the pupil analysis:

*Analysis 3: First exposure*. We calculated the average sound pleasantness rating (on the scale of −5 to +5) for the first exposure to each sound, and compared the U_S_N_V_ and U_S_U_V_ conditions across misophonic and non-misophonic participants (Figure 4). Note that the first exposure corresponds to the U_S_N_V_ movie for participants in Order U_S_N_V_-first, and corresponds to the U_S_U_V_ movie for participants in Order U_S_U_V_-first.

To isolate the effects of the movie condition within the first exposure to sound, the analysis uses a 2 × 2 between-groups ANOVA over items, separating participants based on their Misophonic status (misophonic or non-misophonic) and Movie Type (U_S_U_V_ or U_S_N_V_). A significant main effect was observed for Misophonic status [*F*_(1, 13)_ = 8.878, *p* < 0.05, $\eta_{p}^{2}=0.406$] and Movie Type [*F*_(1, 13)_ = 33.764, *p* < 0.001, $\eta_{p}^{2}=0.722$], and their interaction [*F*_(1, 13)_ = 17.806, *p* < 0.005, $\eta_{p}^{2}=0.578$]. Overall, the misophonic ratings [*M* = −1.73, *SD* = 1.49, Range = (−3.67, 1.83)] were more negative than non-misophonic ratings [*M* = −1.10, *SD*= 1.83, Range = (−4.00, 3.00)]. Between Movie Types, U_S_U_V_ sound ratings [*M* = −2.43, *SD* = 0.91, Range = (−4.00, −0.43)] were more negative than U_S_N_V_ sound ratings [*M* = −0.40, *SD* = 0.91, Range = (−3.67, 3.00)], and this difference was more pronounced for non-misophonic listeners because they rated the movies with neutral video tracks (U_S_N_V_) as more pleasant [*M* = 0.13, 95% CI = (−0.83, 1.08)] than did the misophonic listeners [*M* = −0.92, 95% CI = (−1.82, −0.16); *t*_(13)_ = 3.99, *p* < 0.01]. However, there was no difference in pleasantness ratings for U_S_U_V_ movies between the non-misophonics [*M* = −2.33, 95% CI = (−2.88, −1.77)] and misophonics [*M* = −2.53, 95% CI = (−3.03, −2.03); *t*_(13)_ = 1.04, *p* = 0.32].

##### 2.2.2.1 Analysis 4: repeated exposures

We calculated mean sound pleasantness ratings for each of the repeated exposures to the sounds (Figure 3). This analysis required using sound exposure as a factor (four exposures, because each sound was presented twice with both Movie types). Therefore, in the following analysis, Movie type is not a factor and its effect can be inferred from an interaction between sound Exposure and Order. An ANOVA included factors of sound Exposure number (four exposures), as well as Order and Misophonic status. There was a significant main effect of Misophonic status [*F*_(1, 13)_ = 24.641, *p* < 0.001, $\eta_{p}^{2}=0.655$], and there were two-way interactions between Misophonic status and Order [*F*_(1, 13)_ = 55.35, *p* < 0.001, $\eta_{p}^{2}=0.810$], Exposure and Order [*F*_(1.09, 14.15)_ = 42.667, *p* < 0.001, $\eta_{p}^{2}=0.766$, with Greenhouse-Geisser correction], and a three-way interaction between Misophonic status, Exposure, and Order [*F*_(2.05, 26.65)_ = 4.741, *p* < 0.05, with Greenhouse-Geisser correction]. Below, each of these interactions are considered, in turn.

The main effect of Misophonic status showed that, overall, misophonics rated sounds as less pleasant [*M* = −1.80, 95% CI = (−2.35, −1.24)] compared to non-misophonics [*M* = −1, 05, 95% CI = (−1.65, −0.44)]. A two-way interaction between Misophonic status and Order showed that this difference was greater in the U_S_N_V_-first Order, with misophonic participants giving lower pleasantness rating overall, across all video types [*M* = −2.24, 95% CI = (−2.82, −1.67)], compared to the misophonic participants [*M* = −0.78, 95% CI = (−1.48, −0.09); *t*_(13)_ = 8.39, *p* < 0.001]. However, in the U_S_U_V_-first group, there was no significant difference in the overall mean pleasantness rating between misophonic [*M* = −1.35, 95% CI = (−1.93, −0.77)] and non-misophonic [*M* = −1.31, 95% CI = (−1.85, −0.78)] participants [*t*_(13)_ = 0.21, *p* = 0.84].

The two-way interaction between Exposure and Order reflects the effect of the two video types, N_V_ and U_V_, which was shown more directly as a main effect of Movie Type in Analysis 3. In the U_S_N_V_-first order, the first and fourth exposures corresponded to the U_S_N_V_ movies, and the second and third exposures corresponded to the U_S_U_V_ movies. In contrast, in the U_S_U_V_-first order, the first and fourth exposures corresponded to the U_S_U_V_ movies, and the second and third exposures corresponded to U_S_N_V_ movies. In other words, this interaction is a result of the fact that sounds in the U_S_N_V_ movies were rated as more pleasant [*M* = −0.33, 95% CI = (−1.09, 0.42)] than in the U_S_U_V_ movies [*M* = −2.51, 95% CI = [−3.06, −1.97]; *t*_(13)_ = 6.68, *p* < 0.001], regardless of viewing order.

The three-way interaction between Misophonic Status, Exposure, and Order, can be understood as the effect of misophonia on the *difference* between sound ratings obtained from movies that contained either of the two video types, U_V_ and N_V_. While both misophonics and non-misophonics rated sounds in U_S_N_V_ movies as more pleasant than in U_S_U_V_ movies (as shown in the two-way interaction by Exposure and Order described above), the size of this difference was greater for misophonics [N_V_−U_V_ = 2.35, 95% CI = (1.61, 3.09)] than for non-misophonics [N_V_−U_V_ = 2.01, 95% CI = (1.31,2.72); *t*_(13)_ = 2.26, *p* < 0.05]. Note that this 3-way interaction is the only case (out of all ANOVAs in this study) in which the resampling analysis (2.2.1) using fewer participants did not produce a significant median p-value.

Focused comparisons of same movie conditions. To understand the effects of initial cognitive appraisal and reappraisal on sounds through repeated exposures, we analyzed pleasantness ratings using the same approach applied to pupillary data. Specifically, we compared the same movies across groups in Exposures 1 and 2, as well as the same movies within groups in Exposures 1 and 4.

We compared across order groups to ask whether the sound pleasantness rating upon the first *viewing* of the U_S_U_V_ movie depended on whether the sound was being *heard* for the first time (Exposure 1 in the U_S_U_V_-first order) or following a first exposure to the sound within the U_S_N_V_ movie (sound Exposure 2 in the U_S_N_V_-first order). For misophonic participants, the rating of the first unpleasant video condition was more negative when it followed a neutral video [U_S_N_V_ Exposure 2 - U_S_U_V_ Exposure 1: *M* = −0.842, *SE* = 0.18, *t*_(13)_ = −4.62, *p* < 0.001], whereas for non-misophonic participants, this rating was more positive [U_S_U_V_ Exposure 2 - U_S_N_V_ Exposure 1: *M* = 0.547, *SE* = 0.13, *t*_(13)_ = 4.12, *p* < 0.005]. In summary, a first exposure to an unpleasant sound in the context of a U_S_N_V_ movie has opposite effects on misophonics and non-misophonics, resulting in a more *negative* rating of the sound during the initial (first) viewing of the U_S_U_V_ movie by misophonics but a more *positive* rating of the sound during the initial viewing of the U_S_U_V_ movie by non-misophonics.

In a parallel analysis, we asked whether the sound pleasantness rating upon the initial *viewing* of the U_S_N_V_ movie depended on whether the sound was being *heard* for the first time (sound Exposure 1 in the U_S_N_V_-first order) or following a first exposure to the sound within the U_S_U_V_ movie (sound Exposure 2 in the U_S_U_V_-first order). For misophonic participants, the rating of the initial neutral video condition was more positive when it followed an unpleasant video condition [U_S_U_V_ Exposure 2 - U_S_N_V_ Exposure 1: *M* = 0.63, *SE* = 0.19, *t*_(13)_ = −3.39, *p* < 0.005], whereas for non-misophonic participants, this rating was more negative but not significant [*M* = −0.23, *SE* = 0.32, *t*_(13)_ = 0.73, *p* = 0.48]. In summary, a first exposure to an unpleasant sound in the context of a U_S_U_V_ movie had effects on misophonics but not on non-misophonics, resulting in a more *positive* rating of the sound during the initial viewing of the U_S_N_V_ movie by misophonics.

We next asked whether the sound pleasantness rating was changed by prior viewing of alternative movies *within* participants. During sound Exposures 1 and 4, each participant saw the same movie, either as the first exposure to the sound (which was also the initial viewing of any movie) or as a fourth exposure to the sound (which followed two intervening movies that displayed an alternative source for the sound). Because the type of movie being compared (U_S_U_V_ or U_S_N_V_) was different across test Order, the mean ratings in Exposures 1 and 4 were compared within each test Order. Results within each test Order were separately analyzed for each misophonic and non-misophonic group. Mean ratings did not differ significantly (*p*>0.22 for all) between Exposures 1 and 4 within any of the four groups (misophonics in either test Order and non-misophonics in either test Order). In summary, the pleasantness ratings of the first and last exposure to an unpleasant sound was determined by the movie context it was viewed in during that trial and was unaffected by viewing intervening movies with alternative source interpretations.

## 3 Experiment 2

In Experiment 1, participants were asked to watch movies that included both sound and video tracks and rate the perceived pleasantness of the sound. However, this approach raises two concerns. Firstly, we only recorded valence in terms of pleasantness, without identifying the specific emotions that might have caused it. Secondly, we do not know whether the responses are more related to visual or auditory modalities. To begin to address these concerns, we collected unimodal (visual) data that isolated valence to help identify which specific negative emotions most strongly elicit the observed responses from Experiment 1.

In Experiment 2, we investigated the disgust, anger, and fear emotions that individuals experience when watching silent movies. Participants were asked to rate the U_V_ and N_V_ stimuli without the sounds that were incorporated into the movies in Experiment 1, based on the intensity of disgust, anger, and fear that they evoke. Our primary goal was to explore the correlation between pupil size and visual disgust, as disgust is a significant indicator emotion in misophonic responses to triggering stimuli. By isolating visual stimuli, we want to find additional evidence for whether visual disgust would independently influence physiological responses, including pupil dilation or constriction, which we know can reflect emotional intensity. By comparing the results to the findings from Experiment 1, we expect a negative correlation between visual disgust and pupils based on previous literature that stated visual disgust may constrict the pupil (Ayzenberg et al., 2018; Schienle et al., 2016).

We also measured anger, a strong negative emotion associated with misophonic reactions. Although there is no definitive evidence on whether anger causes pupil dilation or constriction, emotional visual stimuli are generally associated with pupil dilation (Bradley et al., 2008; Murokawa and Nakayama, 2021; Henderson et al., 2018). We also measured fear as a control emotion, as it has negative valence and high arousal, but is not strongly implicated in responses to misophonic triggers.

For pupil data with pleasantness ratings from Experiment 1, we predict a negative correlation between visual disgust and pleasantness, between anger and pleasantness, and between fear and pleasantness. Based on Experiment 1 and the past literature, the first exposure to a sound produces the most significant emotion (Codispoti et al., 2006). Also, Samermit et al. (2022) and Heller et al. (2025) found that the effectiveness of a neutral movie in changing the response to an unpleasant sound was reduced after hearing that sound paired with an unpleasant movie. Therefore, we focus our analysis on the first exposure to each sound. We predict a significant correlation between visual disgust (obtained with the silent movies) and the change in pupil size produced by viewing that movie accompanied by an unpleasant sound.

### 3.1 Methods

#### 3.1.1 Participants

We recruited a new group of 22 participants (*M*_*AGE*_ = 19.2; Range = 18 to 22 years old, 12 females, 9 males, 1 undisclosed) from the student population of Carnegie Mellon University. All participants gave informed consent and were compensated with course credit in accordance with an approved protocol from the CMU Institutional Review Board.

#### 3.1.2 Stimuli

We used the same movies as those in Experiment 1, but removed the sounds.

#### 3.1.3 Procedure

Participants completed the experiment using their personal computers through the Qualtrics platform. The survey began with a set of screening questions to ensure normal hearing. Next, they completed the Duke-Vanderbilt Misophonia Screening Questionnaire (DVMSQ) Williams et al. (2022) to assess individual sensitivity to specific sounds. As we recruited from a general population, all participants were included regardless of their DVMSQ score. One of the 22 participants was misophonic. Next, participants reviewed the instructions and completed a practice trial to familiarize themselves with the task.

The main experiment consisted of 29 silent movies, of which 28 were experimental movies and 1 was a catch trial. The data of participants who failed to correctly answer the catch trials were excluded. The video tracks were presented in randomized orders. After watching each silent movie, participants were asked to rate the intensity of three emotions: disgust, anger, and fear on a 9-point Likert scale (i.e., “How disgusted/angry/afraid did this video make you feel?”) from 1 to 9, with 1 indicating “not at all” and 9 indicating “extremely” (Bradley and Lang, 2000).

### 3.2 Results

The disgust ratings of the silent movies ranged from 1 to 4.27 on a scale from 1 to 9. The mean rating for the U_V_ video tracks was 2.78 (*SD* = 1.00) and the mean for the N_V_ video tracks was 1.24 (*SD* = 0.3).

We correlated the three emotion ratings associated with each of the video tracks (disgust, anger, fear) with the previously obtained sound pleasantness rating (in the context of a movie) from Experiment 1. The correlations were computed over the average value for each movie across all trials. The results show significant negative correlations between sound pleasantness and both disgust [*r* = −0.72, 95% CI = (−0.86, −0.48), *p* < 0.001] and anger [*r* = −0.74, 95% CI = (−0.87, −0.51), *p* < 0.001], but not fear (*r* = −0.29, *p* = 0.13). This result suggests that the perceived unpleasantness of the sounds in this study, heard in the context of movies, is more strongly related to disgust and/or anger than fear. Anger and disgust showed a significant positive correlation with each other [*r* = 0.80, 95% CI = (0.62, 0.91), *p* < 0.001], as did anger and fear [*r* = 0.57, 95% CI = (0.25, 0.78), *p* < 0.01], but there was no significant correlation between disgust and fear [*r* = 0.31, 95% CI = (−0.07, 0.61), *p* = 0.11].

**Figure 5:**
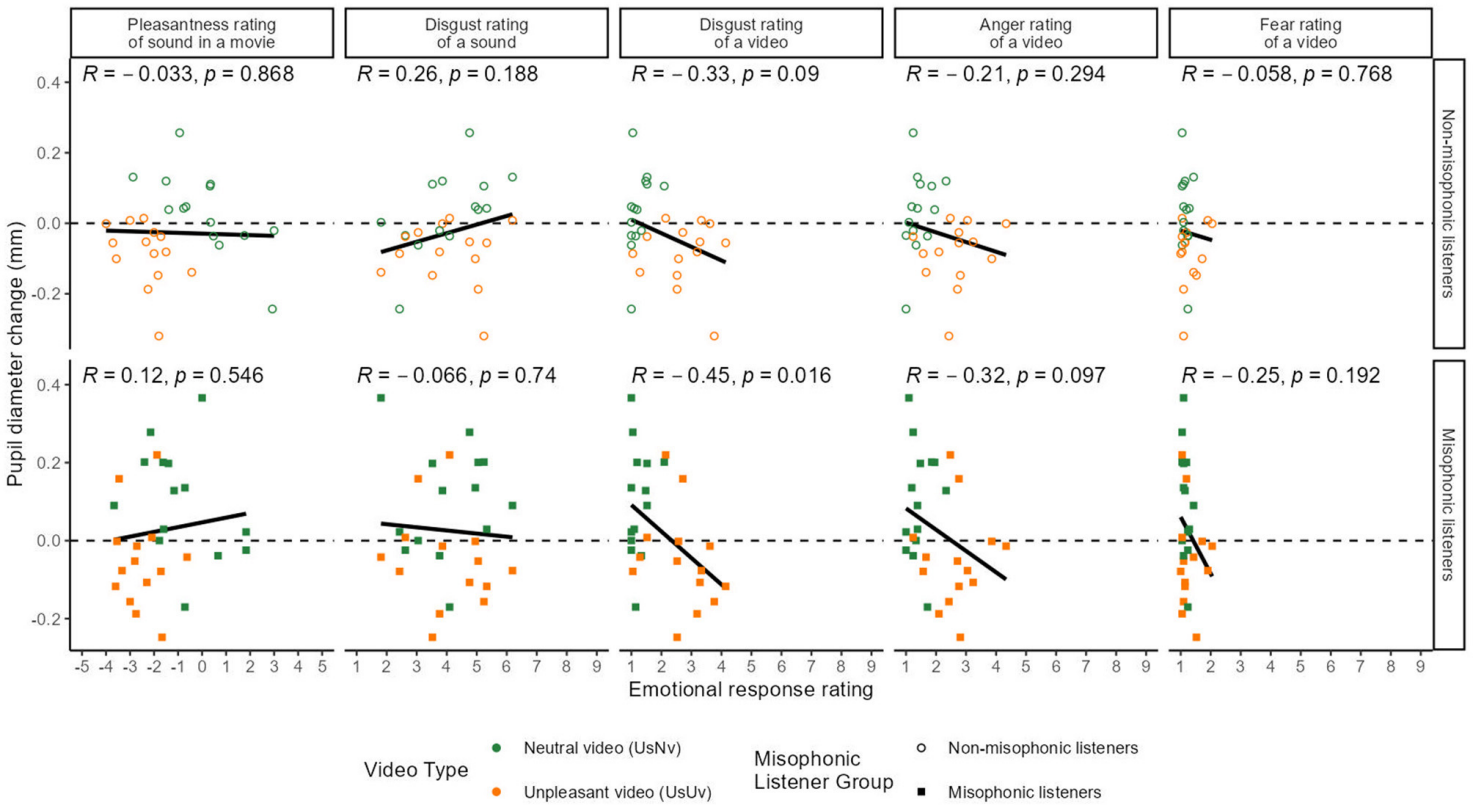
Mean pupil diameter over ratings. The x-axis represents the average ratings and the y-axis represents the mean pupil diameter change from baseline (in millimeters) from 1.5 to 6 seconds. The black line represents the line of best fit. The dashed line (0 mm) indicates no change U_S_U_V_ is displayed in orange and U_S_N_V_ is displayed in green, with each dot representing an averaged value for a single movie. The panels show the pupil response during the initial movie viewing in Experiment 1, correlated with each of five emotional ratings of the stimuli, from left to right: pleasantness rating of the sound (rated in the context of a movie in Experiment 1), disgust toward the sound only (in Experiment 3), disgust rating of the video only (silent movie in Experiment 2), anger rating of the video only (in Experiment 2), and fear rating of the silent movie (in Experiment 2). Results are displayed separately for misophonic and non-misophonic participants.

Next, to understand how specific emotions affect pupil size, we correlated the mean change in pupil diameter during the first exposure of each movie in Experiment 1 to each of the emotion ratings of visual disgust, anger, and fear from Experiment 2 (Figure 5, columns 3–5, respectively) as well as to the sound pleasantness ratings from Experiment 1 (column 1). Given the strong effects of prior contexts on pupils found in Experiment 1, we limited our analysis to the first exposure.

Figure 5 shows that among the eight combinations of participant groups and emotions of the first sound exposure, one significant correlation and one marginal correlation were found between emotion ratings and pupil diameter changes between participants. Specifically, pupil sizes of misophonic participants were significantly correlated to their disgust ratings [*r* = −0.46, 95% CI = (−0.71, 0.10), *p* = 0.014], while pupil sizes of non-misophonic participants were marginally correlated to the movie's disgust ratings [*r* = −0.34, 95% CI = (−0.07, 0.61), *p*= 0.074].

Although we found a significant correlation between disgust and pleasantness ratings and a marginal correlation between disgust and anger ratings, none of the other behavioral ratings (fear, anger, or pleasantness) correlated with pupil size in the first exposure.

## 4 Experiment 3

Experiment 3 was designed to compare unimodal auditory disgust with unimodal visual disgust found in Experiment 2. In this experiment, participants were asked to rate the intensity of disgust, anger, and fear in the sounds previously used in Experiment 1. Our main objective was to explore potential correlations between auditory disgust ratings and pupil responses, to contribute to our understanding of how sound influences pupil reactions observed in Experiment 1.

### 4.1 Methods

#### 4.1.1 Participants

A new group of 21 participants (*M*_*AGE*_ = 21.21, *SD* = 4.47; 12 females, 9 males) were recruited from the student and faculty populations of Carnegie Mellon University. All participants gave informed consent and were compensated in cash in accordance with an approved protocol from the CMU Institutional Review Board.

#### 4.1.2 Stimuli

We used the 14 unpleasant sounds from the 28 movies in Experiment 1.

#### 4.1.3 Procedure

Participants completed the experiment using their personal computers through the Qualtrics platform. The survey began with a set of screening questions to ensure normal hearing. Next, they completed the Duke-Vanderbilt Misophonia Screening Questionnaire (DVMSQ) Williams et al. (2022) to assess individual sensitivity to specific sounds. As we recruited from a general population, all participants were included regardless of DVMSQ score. None of the participants were misophonic. Next, participants reviewed the instructions and completed a practice trial to familiarize themselves with the task.

The main experiment consisted of 14 experimental sounds, plus one catch trial. The data of participants who failed to correctly answer the catch trials were excluded. The sounds were presented in randomized order. After listening to each sound, participants were asked to rate the intensity of the disgust emotion on a 9-point Likert scale (i.e., “How disgusted did this sound make you feel?”) from 1 to 9, with 1 indicating “not at all” and 9 indicating “extremely” (Bradley and Lang, 2000).

### 4.2 Results

The average disgust rating of the sounds was 4.05 (*SD*=1.27) with a range from 1.81 to 6.19, on a scale from 1 to 9. We found that the disgust of the sound alone correlated significantly with the disgust of the silent movie in Experiment 2 [*r* = 0.40, 95% CI = (0.03, 0.67), *p* < 0.05] and with the pleasantness of the sound rating in the context of a video track from Experiment 1 [*r* = −0.50, 95% CI = (−0.74, 0.16), *p* < 0.01]. However, in contrast to the ratings of purely visual disgust in Experiment 2, the ratings of purely auditory disgust in this experiment did not significantly correlate with pupil diameter changes in the first exposure of Experiment 1 for either misophonic [*r* = −0.07, 95% CI = (−0.43, 0.32), *p* = 0.74] or non-misophonic [*r* = 0.26, 95% CI = (−0.13, 0.57), *p*= 0.19] listeners (as shown in the second column of Figure 5). Unimodal auditory-disgust did not correlate with pupil diameter changes in any exposure.

## 5 Discussion

This study explored the effects of misophonia on pupillary responses to auditory-visual stimuli, with a focus on how visual reinterpretation of sounds influences emotional and physiological responses. Across the three experiments, we collected behavioral ratings for auditory-visual, visual-only, and auditory-only stimuli. Our results revealed that the visual context of sounds significantly affects emotional and pupillary responses. Ratings and pupil responses during the first exposure to a sound followed a similar pattern: a neutral video track paired with an unpleasant sound led to pupil dilation and higher pleasantness ratings, while an unpleasant video track paired with the same sound led to pupil constriction and lower pleasantness ratings. Furthermore, pupil responses from Experiment 1 correlated with the perceived disgust toward the video tracks in Experiment 2, highlighting the correlation between physiological and emotional reactions, but they did not correlate with the perceived auditory disgust toward the sound in Experiment 3. Although disgust had explanatory power for the audiovisual stimuli while anger did not, this does not deny the role that anger and other emotions (such as irritation) can play in the experience of misophonia; rather, it shows that disgust, given its unique pupillometry signal, can be a useful marker of intense emotional responses to misophonic/unpleasant movies for both misophonic and non-misophonic observers. These findings, along with previous work showing that auditory stimuli alone typically dilate the pupil—including negative emotions like disgust (Oszczapinska et al., 2025)—suggest that visual stimuli played an important role in shaping the pupillary responses.

Firstly, during the first sound exposures in Experiment 1, the U_S_N_V_ ratings were higher than U_S_U_V_ ratings. This confirms that unpleasant sounds accompanied by neutral-emotion video tracks are initially perceived as more pleasant than when accompanied by their original unpleasant video track. In Experiment 2, we collected ratings for three specific emotions—disgust, anger, and fear—from the same video tracks. There was a significant correlation between pleasantness, disgust, and anger, but not between pleasantness and fear. The absence of correlation with fear validates our prediction that misophonic triggers specifically evoke disgust and anger instead of fear. This observation is consistent with previous research that identified anger and disgust as the primary emotional responses to misophonic triggers (Ferrer-Torres and Giménez-Llort, 2022). As a result, the unpleasant ratings evoked from the movies can be attributed to disgust and anger. In Experiment 1, the group with misophonia exhibited increased negative emotions to unpleasant sounds in our experimental context, as predicted. Nonetheless, data for both groups agreed that neutral video tracks alleviate negative emotional responses to unpleasant sounds.

Secondly, for the first exposure, we explored pupil dilation and constriction in response to misophonic triggers for misophonic and non-misophonic individuals. As a reminder, we restricted Analysis 1 of Experiment 1 to only the first exposure of each sound so that the results would not be affected by prior exposure or order. That is, we separately examined the U_S_N_V_ condition for the U_S_N_V_-first order participants and the U_S_U_V_ condition for the U_S_U_V_-first order participants. When the first exposure was the U_S_N_V_ condition, pupil sizes increased from baseline overall, with this pupil dilation being reliable for misophonic participants. Previous research has associated increased pupil size to more intense emotional responses to visual and auditory stimuli separately (Babiker et al., 2014; Bradley et al., 2008; Henderson et al., 2018; Murokawa and Nakayama, 2021; Partala and Surakka, 2003; Partala et al., 2000). In contrast, when the first exposure was the U_S_U_V_ condition, pupil sizes decreased from baseline overall for both misophonic and non-misophonic groups. This result aligns with the findings by Ayzenberg et al. (2018), who found pupil constriction in response to specifically visual disgust stimuli. Given that misophonic triggers are often associated with disgust, and given that our ratings of unpleasantness highly correlated with disgust in our study, our data support this connection. The visual component of the movie must be causing the pupil sizes in the U_S_N_V_ movies to be larger than those in the U_S_U_V_ movies because the sound was the same in both movies. In summary, our findings suggest that auditory-visual stimuli associated with visual disgust constrict the pupil, whereas neutral auditory-visual stimuli dilate the pupil. Therefore, pupillary responses could be a good indicator of auditory-visual disgust, which could inform clinical approaches to treating misophonia. Monitoring temporal changes in pupil size, particularly with visual interventions aiming to alleviate the effects of trigger sounds, may reveal novel strategies to mitigate the unpleasant emotional responses associated with misophonia triggers.

In the first exposure, when we related pupil responses (in Experiment 1) to behavioral ratings of the stimuli (across Experiments 1, 2, and 3), we found that disgust was the only reliable predictor of pupil diameter. It is important to note that the disgust, anger, and fear ratings were collected from different participants (in Experiments 2 and 3) and, therefore, introduced between-participant variability in emotional responses. These studies used separate participants so that the effects of visuals could be isolated from the effects of sounds. The pleasantness ratings, on the other hand, were obtained in Experiment 1 from the same participants whose pupil diameters were being measured, so if participant variability was the determining factor, these ratings would be expected to correlate most highly with individual pupil responses. Despite the fact that our disgust, anger, and fear measurements were from mostly non-misophonic participants, we found that visually-evoked disgust correlated significantly with misophonic pupil diameter changes in Experiment 1, whereas the pleasantness of sounds (comparing within misophonic groups) did not. Therefore, variability across participants and groups was small enough that it was still possible to discover a relationship between emotional and physiological responses.

Thirdly, examining rating data across repeated exposures, we found that the beneficial effect of a neutral video on emotional ratings was robust across repeated exposures (with no main effect of test Order). Although misophonic participants gave lower ratings in the U_S_N_V_-first order (in an interaction between Order and Misophonic status), across both misophonic and non-misophonic groups, U_S_N_V_ movies were consistently rated as more neutral and significantly less negative than U_S_U_V_ movies. Furthermore, Exposure had a greater impact on rating differences between movie types in the U_S_N_V_-first order than in the U_S_U_V_-first order (in an interaction between Order and Exposure). Overall, these findings show that pairing unpleasant sounds with neutral videos (U_S_N_V_) improves perceived pleasantness of the sounds across all four exposures and for both participant groups. The neutral videos appear to protect listeners from experiencing the most intense negative emotions triggered by the sounds. This implies that neutral video pairings may be an effective intervention strategy to reduce emotional distress from triggering sounds, regardless of exposure order and repetition (up to four exposures in this study). To our knowledge, this is the first empirical presentation of such a protective effect persisting across many repetitions.

Fourthly, examining pupil data across repeated exposures, we found pupil diameters to be a meaningful indicator of emotional response during the first exposure, but less predictive in subsequent exposures. While pupil diameter and ratings are correlated in the first exposure, subsequent exposures diverge. We explored the effects of repeated exposures to the same sound in different visual contexts, as well as repeated viewings of the same movie, on both emotional and physiological responses. The first effect of repeated exposures we addressed was whether pupillary and behavioral responses upon the initial, naive viewing of a movie depended on whether the sound was also being heard for the first time, or if there had already been a first exposure to the sound within the opposing movie condition. On the one hand, we found that when misophonic participants first watch the U_S_U_V_ movie after initially watching the U_S_N_V_ movie (compared to not watching U_S_N_V_ previously), their pupils constrict less (i.e., show less change from baseline), and they rate U_S_U_V_ movies as more unpleasant. However, for non-misophonic participants under the same conditions, their pupils don't reliably differ, and they rate U_S_U_V_ movies as less unpleasant. On the other hand, when misophonic participants first watch the U_S_N_V_ movie after previously watching the U_S_U_V_ movie once (compared to not watching U_S_U_V_ previously), their pupils dilate less, and they rate U_S_N_V_ movies as more pleasant. Similarly, for non-misophonic participants under the same condition, their pupils also dilate less, but they don't rate U_S_N_V_ movies differently. Through these findings, we found that the first naive viewing of a movie creates the largest pupil response. After a sound is heard once, pupil response is reduced (closer to neutral) in exposure 2. This effect is observed in both video types for misophonic participants, and in the U_S_N_V_ video type for non-misophonic participants. Additionally, while the first naive viewing of a movie also gives the largest behavioral response in some conditions, there is one condition with the opposite result: misophonics give a more negative rating to the sounds in an unpleasant movie (U_S_U_V_) when it is their second time hearing the sound (even though the first exposure is with a neutral movie).

The second effect of repeated exposures we addressed was whether the pupillary and behavioral responses of a *repeated* movie were changed *within* participants by a prior viewing of alternative movies. We found no significant effects of the intervening movies, for either misophonic or non-misophonic groups. This was true whether the first and fourth exposure were to the U_S_U_V_ movie or the U_S_N_V_ movie. These findings indicate that the emotional reappraisal of sound pleasantness in the context of a movie (i.e., rating the sound as more pleasant in the context of a neutral movie than in the context of an unpleasant movie) is just as effective when viewing the movie repeatedly (initial vs. second viewing), and is unaffected by intervening repeated exposures to the sound in the context of movies with different interpretations of the sound source. Of particular relevance to treatment considerations, this means that, based on behavioral ratings, a neutral movie that makes an unpleasant sound more bearable will still make it more bearable upon repeated exposures, even after seeing an unpleasant movie. This conclusion is limited to what happens upon repeated viewings once a movie has been initially viewed, because the initial viewing of a neutral movie may be affected by prior exposures (indicated by the comparison of Exposures 1 vs. 2, above). Of complementary relevance to treatment, behavioral ratings of a sound in an unpleasant movie may continue to be unpleasant in repeated exposures, even after seeing an intervening neutral movie. Note that, overall, the pattern of pupil responses with repeated exposures does not mirror the pattern of behavioral ratings. However, specifically comparing the pupil responses to Exposures 1 and 4, the pupil diameter is not reliably different, which mirrors the lack of behavioral differences seen between the first and last exposure.

Overall, these findings suggest that repeated viewing of a movie does not significantly affect either pleasantness ratings or pupil diameter. Our findings support that U_S_N_V_ videos can serve as effective interventions, and we can use ratings as reliable indicators of individuals' emotional experiences. Negative U_S_U_V_ videos should be avoided in therapeutic contexts and used only in controlled research settings, such as the present one. Practitioners using pupils as an adjunct measure should consider that pupil response is very sensitive to recent exposures. Without understanding a patient's prior exposures, pupil size alone is insufficient to predict emotional responses and should not replace emotional ratings. However, pupillometry may prove more robust to prior exposures at longer time frames. Currently, we suggest that pupillometry can be useful upon first exposure to gauge the effectiveness of particular U_S_N_V_ videos, with average responses of pupil dilation (as opposed to constriction) potentially revealing that a particular U_S_N_V_ video is effective. We do not have enough data to say whether this approach would work at the individual level, but this application could be the eventual goal. However, this would only be recommended as an adjunct to the emotional rating, which reflects the conscious experience.

Fifthly, we specifically examined our findings on disgust. We initially explored the relationship between pupil diameter across all exposures. However, we decided to focus only on the first exposure, as for the remaining exposures, it was unclear whether the reactions were from the current condition or influenced by previous trials. In the first exposure, among the three types of emotional categories (disgust, anger, fear) and valence (pleasantness), only disgust showed a significant correlation with pupil diameter. Furthermore, this correlation was in the predicted direction (negative). These findings suggest that participants in Experiment 1 showed greater pupil constriction to more visually disgusting stimuli, as rated by the sample of mostly non-misophonic participants in Experiment 2 (significantly for misophonics, marginally for non-misophonics in Experiment 1). Furthermore, pupil diameter was not correlated with either the sound pleasantness in the context of the movie or the disgust toward the sound alone, but it was correlated with visual disgust toward the video alone. Taken together, these results imply that visual disgust is the dominant emotional influence on pupil responses to these auditory-visual stimuli. Moreover, these results suggest that studying responses to disgust in a general population is not an unreasonable way to study disgust in misophonia.

Previous studies have shown that auditory disgust generally causes pupil dilation. Our data agree with pupil constriction in response to visual disgust. There is no prior result for auditory-visual disgust to compare with this finding. Additionally, we ruled out purely low-level visual explanations for our findings, such as brightness or spatial frequency, for our effect. Visual factors were ruled out by the fact that the initial pupil response to a neutral movie is different from the response to it upon second viewing (after seeing the unpleasant interpretation of the sound). Based on these observations, we propose that the pupillary responses in our data were likely influenced by auditory-visual interactions and contextual factors, including memory of past associations, which might not be reflected in the emotional ratings. However, since we did not include a video track-only condition for the participants in Experiment 1, we cannot determine whether auditory-visual pairing was essential for this finding. Future studies should address this gap.

The findings of this study may help to develop methods to monitor individual progress in misophonia treatment by tracking physiological responses, such as pupil size. Monitoring changes in pupil size over time could provide a more objective way to evaluate the effectiveness of treatments that aim to reduce the emotional distress triggered by misophonic stimuli. Currently, effective treatments with some supporting evidence include counterconditioning (Dozier, 2015), Cognitive Behavioral Therapy (CBT) (Beck and Fernandez, 1998; Bernstein et al., 2013), and Tinnitus Retraining Therapy (TRT) (Jastreboff and Jastreboff, 2014). These approaches use self-reported responses and several physiological responses, such as galvanic skin conductance and heart rate, to monitor therapy progress. They could benefit from incorporating pre-test and intermittent post-test assessments to additionally track pupillary responses, providing a more comprehensive evaluation of treatment efficacy.

Lastly, to expand and diversify the available stimuli, researchers should focus on presenting each type of trigger sound in more varied contexts. For example, they could instead use simpler media, such as words and images, to present stimuli in different settings, as recommended by Heller et al. (2025). To better reflect real-world variability, researchers could also generate multiple recordings per sound type (e.g., several crunching samples). Furthermore, recent advances in AI-generated sounds provide a promising tool to efficiently build large databases of trigger sounds. Moreover, clinicians might experiment with pairing pleasant videos with foley-generated misophonic sounds to match the video timing (Du et al., 2023; Lee et al., 2024), which could simplify the movie creation process.

## 6 Conclusion

We found evidence suggesting that visual disgust from mildly disgusting movies can lead to pupil constriction. Based on the different movie conditions in the first exposure, which shared the same auditory condition but had different video tracks, we found that the amount of pupil constriction in misophonic participants was quantitatively related to how disgusting the visual stimuli were. Furthermore, we observed pupil dilation in response to unpleasant sounds when paired in a movie with a neutral video track.

The pattern of findings was mostly consistent for both misophonic and non-misophonic individuals. However, misophonic participants showed a larger pupil size difference between the two types of movies, consistent with the fact that misophonic individuals rated the misophonic sounds in the context of the movies as more unpleasant than the non-misophonic individuals.

People with misophonia and their families may benefit from knowing that the physiological and emotional response to a given sound is not fixed in stone, because a different response can be evoked when that sound is cognitively reinterpreted. There is hope that non-invasive physiological monitoring may produce advances in the ability to objectively evaluate whether a treatment is effective. Researchers will benefit from knowing that the physiological response of pupils narrowing when viewing misophonic movies is similar across young adults whether or not they are misophonic, which means that the pupil's physiological response does not determine the emotional response and may even follow it. In this respect, pupillometry could be a useful indicator of when the emotional response to a trigger has lessened. Researchers should investigate the duration of the effect of prior exposure to a movie (i.e., a sound source interpretation for a given sound) so that they can derive the optimal benefit from source reinterpretation, perhaps by spacing out the exposures and varying the stimuli. Clinicians should benefit from understanding that the physiological response to the very first exposure during each therapy session is likely to be unique; this will improve monitoring of treatment effectiveness.

## Data Availability

The original contributions presented in the study are publicly available. This data can be found here: https://doi.org/10.1184/R1/c.7112221.

## Footnotes

^1^ https://www.cmu.edu/inclusive-excellence/tartans/index.html

^2^ https://doi.org/10.1184/R1/c.7112221

^3^ https://www.apple.com/imovie/

^4^ https://github.com/hellerl/Misophonia/tree/main/Image%20Extract%20and%20Distort
